# Sonic hedgehog signalling pathway in CNS tumours: its role and therapeutic implications

**DOI:** 10.1186/s13041-024-01155-w

**Published:** 2024-11-20

**Authors:** Andrew Awuah Wireko, Adam Ben-Jaafar, Jonathan Sing Huk Kong, Krishitha Meenu Mannan, Vivek Sanker, Sophie-Liliane Rosenke, Allswell Naa Adjeley Boye, Princess Afia Nkrumah-Boateng, Jeisun Poornaselvan, Muhammad Hamza Shah, Toufik Abdul-Rahman, Oday Atallah

**Affiliations:** 1 Faculty of Medicine, Sumy State University, Sumy, 40007 Ukraine; 2 School of Medicine, University College Dublin, Belfield, Dublin 4, Ireland; 3 School of Medicine, College of Medical & Veterinary Life Sciences, University of Glasgow, Glasgow, UK; 4 School of Medicine, Dentistry & Biomedical Sciences, Queen’s University Belfast, Belfast, UK; 5 Department of Neurosurgery, Stanford University, Stanford, CA USA; 6 School of Medicine, Lancaster University, Lancaster, UK; 7 University of Ghana Medical School, Accra, Ghana; 8 Department of Neurosurgery, Hannover Medical School, Carl-Neuberg-Strasse 1, 30625 Hannover, Germany

**Keywords:** Sonic Hedgehog signalling pathway, Brain tumours, Molecular neuro-oncology, Neuro-genetics

## Abstract

CNS tumours encompass a diverse group of neoplasms with significant morbidity and mortality. The SHH signalling pathway plays a critical role in the pathogenesis of several CNS tumours, including gliomas, medulloblastomas and others. By influencing cellular proliferation, differentiation and migration in CNS tumours, the SHH pathway has emerged as a promising target for therapeutic intervention. Current strategies such as vismodegib and sonidegib have shown efficacy in targeting SHH pathway activation. However, challenges such as resistance mechanisms and paradoxical effects observed in clinical settings underscore the complexity of effectively targeting this pathway. Advances in gene editing technologies, particularly CRISPR/Cas9, have provided valuable tools for studying SHH pathway biology, validating therapeutic targets and exploring novel treatment modalities. These innovations have paved the way for a better understanding of pathway dynamics and the development of more precise therapeutic interventions. In addition, the identification and validation of biomarkers of SHH pathway activation are critical to guide clinical decision making and improve patient outcomes. Molecular profiling and biomarker discovery efforts are critical steps towards personalised medicine approaches in the treatment of SHH pathway-associated CNS tumours. While significant progress has been made in understanding the role of the SHH pathway in CNS tumorigenesis, ongoing research is essential to overcome current therapeutic challenges and refine treatment strategies. The integration of molecular insights with advanced technologies and clinical expertise holds great promise for developing more effective and personalised therapies for patients with SHH pathway-driven CNS tumours.

**Graphical Abstract:**

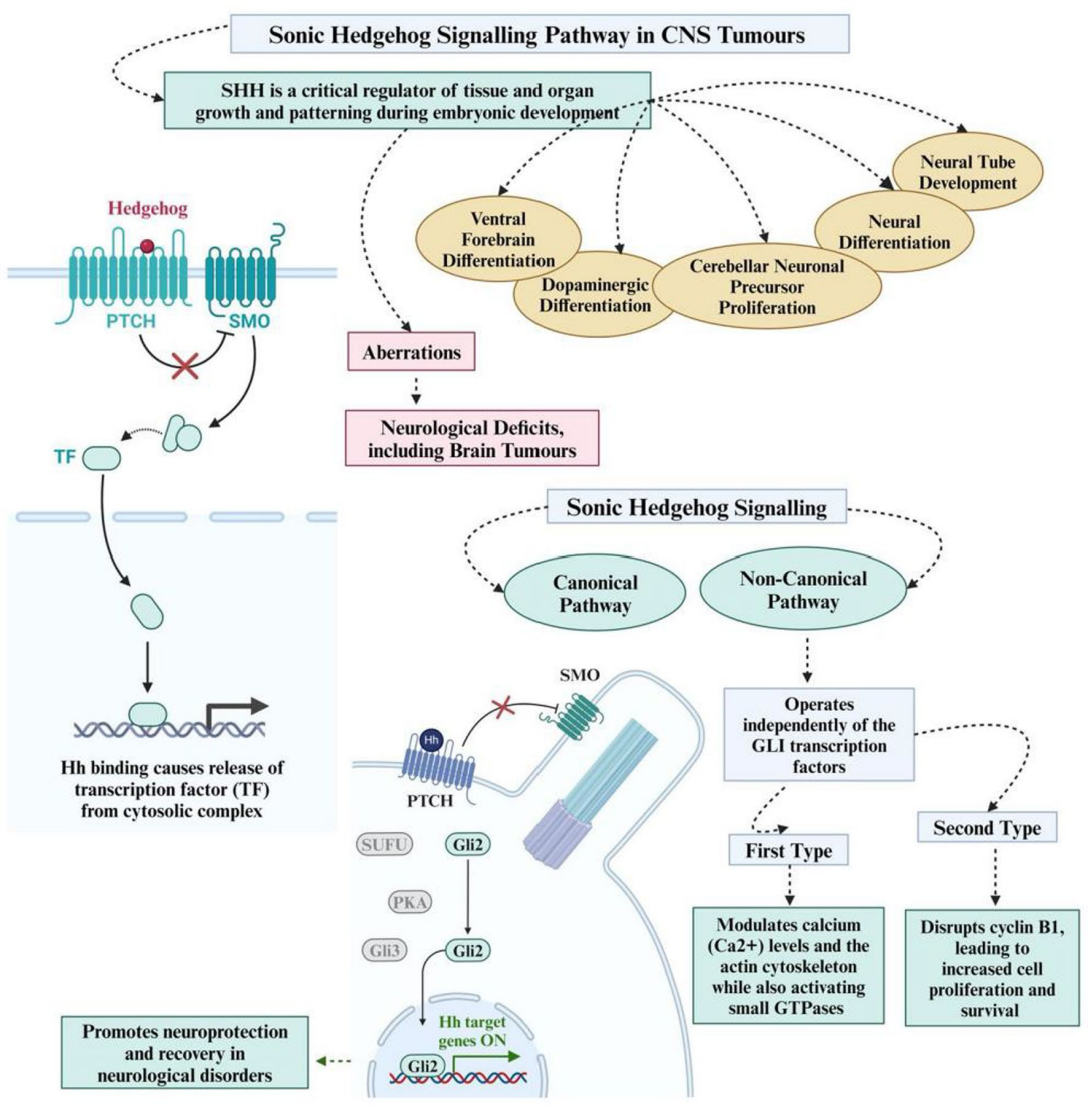

## Introduction

Central nervous system (CNS) tumours are abnormal cell developments in the brain, spinal cord, intracranial endocrine glands and other parts of the CNS [1]. Compared to other malignancies, CNS tumours are rare in adults, but they represent a significant burden of morbidity and mortality [2]. The latest Global Cancer Observatory (GLOBOCAN) report shows that CNS tumours ranked 19th out of 321,731 cancer cases and 12th out of 248,500 cancer deaths worldwide. In terms of incidence rates, most cases of CNS tumours are found in Asia with 177,139 (55.1%) of the global incidence, followed by Europe with 67,559 (21.0%), North America with 28,126 (8.7%), Latin America and the Caribbean with 26,992 (8.4%), Africa with 19,289 (6.0%) and Oceania with the least incidence of 2626 (0.82%) [3]. Asia also has the highest CNS tumour mortality rate at 132,799 (53.4%) of the global mortality rate, with Oceania having the lowest reported mortality rate at 0.80% [3]. Due to the complex nature of most CNS tumours, the diagnosis and treatment of these tumours in the clinical setting has been a major challenge for many decades.

However, there have been tremendous advances in molecular trends in CNS tumour research aimed at improving the diagnosis and treatment of these tumours. Some of these new trends include the discovery of the 1p/19q marker for oligodendroglial tumours and intradialytic hypotension (IDH) mutations in gliomas as an important factor in the classification of diffuse gliomas on a molecular basis, the telomerase reverse transcriptase (TERT) promoter has been identified as an important factor in telomere lengthening and CNS tumorigenesis, and triggering receptor expressed on myeloid cells (TREM) has emerged as a potential target to assess the tumour immune microenvironment [4–7]. The HH signalling pathway has also been found to be widely involved in CNS malignancies, making it a potential target for cancer therapy. In addition to regulating cancer cell properties, the HH pathway has been shown to play an immunoregulatory role within the tumour microenvironment (TME) [8].

The sonic hedgehog (SHH) signalling pathway, an example of HH, is a critical regulator of tissue and organ growth and patterning during embryonic development. This unique pathway is strongly associated with neural tube development, neural differentiation, patterning of the ventral forebrain, dopaminergic differentiation of the midbrain, proliferation and survival of ventral progenitors, proliferation of cerebellar neuronal precursors and patterning of the developing thalamus [9]. Aberrations in the SHH pathway therefore lead to neuronal degeneration and various neurological deficits, including brain tumours. Activation of the SHH pathway occurs in two main mechanisms: canonical and non-canonical signalling. Canonical signalling is initiated when the SHH ligand binds to the Patched (PTCH) receptor at the cell membrane. Normally, PTCH inhibits the Smoothened (SMO) protein, but ligand binding releases this inhibition, allowing SMO to activate the downstream signalling cascade [6]. This activation by the 7-transmembrane protein SMO promotes neuroprotection and recovery in neurological disorders. Non-canonical signalling, on the other hand, operates independently of the GLI transcription factors and can be divided into two types. The first type modulates calcium (Ca^2^ +) levels and the actin cytoskeleton while also activating small GTPases such as RhoA and Rac1 [6]. The second type, SMO-dependent type II signalling, disrupts cyclin B1, leading to increased cell proliferation and survival [6].

The aim of this review is to explore the role of the SHH pathway in CNS tumorigenesis, thereby providing insight into defective pathways and facilitating the development of effective therapeutic interventions for CNS malignancies.

## Methods

This narrative review aims to provide a comprehensive framework of the role of the SHH pathway in CNS tumours. Specific inclusion and exclusion criteria were used to ensure a rigorous and comprehensive approach. The inclusion criteria consisted of full-text articles written in English. Several databases were used, including PubMed/Medline, EMBASE, the Cochrane Library and Scopus. Key words such as 'sonic hedgehog', 'SHH', 'CNS tumours', 'gliomas', 'meningiomas', 'medulloblastomas' and 'neuroblastomas' were used for a comprehensive database search. References cited in recent reviews on similar topics were also manually reviewed to identify additional sources that could contribute to the search strategy. Standalone abstracts, conference proceedings, letters to editors, editorials, perspectives and posters were excluded, with priority given to the inclusion of high quality and reliable evidence. In addition, the review did not limit the publishing dates and the number of studies to provide a comprehensive manuscript. It included descriptive, animal model, cohort and observational studies from both preclinical and clinical settings to provide a holistic perspective. A summary of the methodology used is shown in Table 1.

**Table 1:** Summary of methodology

Methodology steps	Description
Literature search	PubMed/MEDLINE, EMBASE, Scopus and the Cochrane Library
Inclusion criteria	Various study designs including experimental studies, randomised controlled trials, prospective and retrospective cohort studies Studies involving both paediatric and adult populations Studies providing raw data Full-text articles published in English
Exclusion criteria	Non-English studies, stand-alone abstracts, conference proceedings, editorials, commentaries, and letters
Search terms	Key words such as 'sonic hedgehog', 'SHH', 'CNS tumours', 'gliomas', 'meningiomas', 'medulloblastomas' and 'neuroblastomas' were used for a comprehensive database search
Additional search	A manual search was performed to include references from recently published procedure-specific and disease-specific reviews
Sample size requirement	No strict sample size requirement

## SHH pathway in CNS development

SHH is a glycoprotein that functions as a critical signalling molecule in the development of the neural tube (NT), which gives rise to the brain and spinal cord [10]. The NT, the embryonic precursor of the CNS, forms through gastrulation during early embryonic development [11]. Defects in neural tube development (NTD) are among the most common birth defects in humans [12].

The CNS development begins with the folding of the posterior neural plates, guided by molecular signals from the notochord and prechordal mesoderm, leading to the formation of the NT by 3 to 4 weeks post-conception. Neurulation, divided into primary and secondary phases, involves the closure of the anterior and posterior neuropores, forming the brain and spinal cord [13]. The SHH pathway regulates NT formation by controlling the patterning of the NT and providing signals to ventral neural progenitors during neurogenesis [11]. Absence of SHH leads to serious midline defects such as holoprosencephaly, with associated cardiac and genitourinary anomalies [14, 15]. Elevated SHH signalling is linked to exencephaly, anencephaly, encephalocoele, and spina bifida, due to the incomplete closure of the spinal cord and backbone [16–18].

SHH plays a crucial role in neural stem cell (NSC) development. NSCs, derived from the neural crest, are multipotent cells capable of self-renewal and differentiation into neuronal and glial subtypes [19]. During early brain development, NSCs in the ventral zone increase in number through even division, followed by asymmetric division during neurogenesis to produce NSCs and neurons. As gestation progresses, NSCs generate astrocytes, oligodendrocytes, and neurons, marking the end of the neurogenic phase [20].

The SHH pathway controls NSC proliferation in vivo, shortening their time in the G1 and S-G2/M phases. Excessive activation can lead to the accumulation of quiescent NSCs, impairing neuronal development. Thus, SHH is vital for maintaining CNS homeostasis and proper development during injury [21]. In cerebellar development, SHH mediates the interplay between Purkinje cells (PCs) and Granule Cell Progenitors (GCPs), regulating GCP proliferation and ensuring proper cerebellum size and foliation [22]. SHH also inhibits cell apoptosis and inflammation via the *Nurr1* gene, maintaining interneuron activity in the medial ganglionic eminence and aiding oligodendrocyte progenitor cell production [22–25].

## The SHH signalling pathway components/classifications in tumorigenesis

### The canonical pathway

The canonical SHH pathway is a conserved signalling cascade crucial for embryonic development and tissue patterning [26]. Encoded by the SHH gene, the *45-kDa* precursor protein transcribes into the SHH ligand, the primary signalling molecule initiating the pathway by binding to PTCH1 receptors [22]. PTCH1 is distributed in the primary cilia and in absence of SHH, PTCH1 inhibits the pathway, by inhibiting another protein, SMO, preventing downstream signalling, maintaining the pathway in an inactive state. When SHH binds to PTCH1, it leaves the cilia and its inhibition of SMO is lifted, activating a canonical SHH signalling cascade in the primary cilia and allowing SMO to activate downstream signalling components [22]. The signally pathway involves two steps: ciliary localization and subsequent activation. The interactions between PTCH1 and SMO are argued to be mediated by accessible (membrane) cholesterol [22].

Besides PTCH1, other SHH co-receptors, such as CDON, BOC, and GAS1 are essential for SHH pathway activation and are also involved in CNS development. This includes cell fate specification, axon guidance, and cell proliferation [22]. This cascade leads to SMO reaching its major target, which is regulation of GLI family transcription factors, specifically GLI1, GLI2, and GLI3. GLI1 primarily acts as a transcriptional activator, promoting the expression of target genes involved in cell proliferation and survival. GLI2 can function as both an activator and a repressor, while GLI3 generally acts as a repressor. When activated, GLI1 and GLI2 promote the expression of target genes involved in cell proliferation, survival, and differentiation [26].

In absence of SHH signalling, the GLI proteins are inhibited by Suppressor of Fused (SUFU) by sequestering GLI in the cytoplasm. Kinesin family member 7 (KIF7) is an additional protein regulating the GLI proteins activity and localisation in conjunction with SUFU [27]. On stimulation by SHH, the SUFU-GLI complex dissolves from the tip of the cilia, as the GLI dissociates from SUFU and translocates to the nucleus, activating the SHH pathway [22].

### The non-canonical pathway

**Fig. 1:**
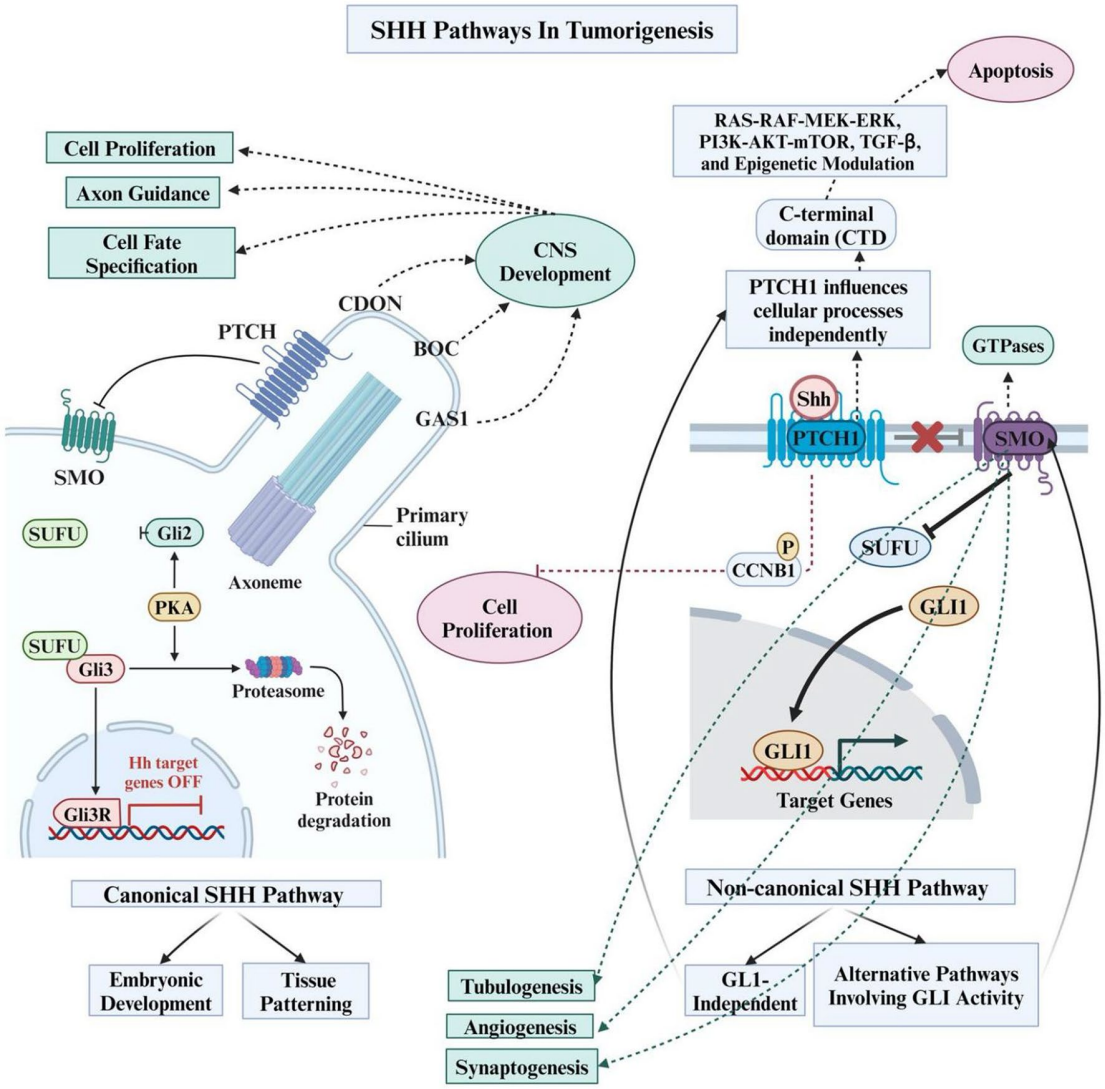
The SHH pathways in tumorigenesis and their types (canonical and non-canonical). Image was created with https://www.Biorender.com. CNS; Central Nervous System, CDON; Cell Adhesion Associated Oncogene Regulated; BOC; Butoxycarbonyl, GAS; Growth Arrest Specific, PTCH; Patched, SMO; Smoothened, SUFU; Suppressor of Fused; PKA; Protein Kinase A, CTD; C-Terminal Domain, CCNB; Cyclin B, GTPase; Guanosine Triphosphate, SHH; Sonic Hedgehog, RAS; Reticular Activating System, RAF; Rapidly Accelerated Fibrosarcoma, MEK; Mitogen-Activated Protein, ERK; Extracellular-Signal-Regulated kinase, PI3K; Phosphatidylinositol-3 Kinase, AKT; Protein Kinase B, mTOR; Mammalian Target of Rapamycin, TGF-β; Transforming Growth Factor Beta

The non-canonical SHH pathway represents alternative signalling routes from the canonical pathway, providing additional complexity and flexibility and insight into cellular responses and tumorigenesis. It encompasses two types: (1) GLI-independent; and (2) alternative pathways involving GLI activity. While SMO plays a crucial role in the canonical pathway, the non-canonical SHH pathway can independently activate downstream effects without SMO involvement, mainly through GLI activity [28]. In type 1, PTCH1 influences cellular processes independently of SHH, PTCH1-SMO interactions, or GLI transcription factors. The PTCH1 C-terminal domain (CTD) induces apoptotic cell death via alternative pathways like RAS-RAF-MEK-ERK, PI3K-AKT-mTOR, TGF-β, and epigenetic modulation [26]. PTCH1 also negatively regulates cell proliferation by interacting with phosphorylated CCNB1, a G2/M checkpoint regulator [22]. Type 2 is SMO-dependent, and SMO has shown to not only function as a classic SHH signalling transducer, but is also responsible for activating small GTPases, such as Rac1 and RhoA and rearranging the actin cytoskeleton for the proper regulation of cell processes, such as angiogenesis, tubulogenesis and synaptogenesis [22]. The SHH pathways in tumorigenesis have been summarised in Fig. 1.

## The role of SHH signalling pathway in CNS tumours

### Gliomas

Gliomas, originating from glial cells in the brain and spine, represent a diverse group of tumours known for their aggressive behaviour and resistance to treatment, posing significant therapeutic challenges. The SHH signalling pathway, a crucial regulator of cellular proliferation, differentiation, and tumorigenesis, has been increasingly recognized for its role in the pathogenesis of CNS tumours [29]. SHH signalling influences glioma growth and development through various mechanisms. The tumour stroma, mainly consisting of endothelial cells, adipocytes, immune cells, and cancer-associated fibroblasts (CAFs), secretes soluble factors that promote tumour metastasis and chemotherapy resistance [30, 31]. Recombinant human SHH N-terminal peptide (rhSHH) enhances HH signalling, leading to increased mRNA levels of matrix metalloproteinase-2 *(MMP2)* and *MMP9*, which facilitate the adhesion and invasion of GBM cells [31].

Additionally, SHH signalling in GBM cells is significantly amplified by Fms-related tyrosine kinase 1 (*FLT1*). Suppressing SHH signalling reduces the migration and invasion driven by FLT1 overexpression, whereas enhancing SHH signalling restores FLT1's invasive and migratory capabilities [32]. FLT1, a tyrosine kinase receptor that binds VEGF-A, promotes tumour growth and metastasis through angiogenesis [32, 33]. Moreover, truncated *GLI1 (TGLI1),* a product of SHH signalling, functions as an enhanced *GLI1*, promoting angiogenic heparanase expression and thereby facilitating GBM angiogenesis and tumour growth [34]. *GLI2*, another SHH signalling product, is stabilised by *mTORC2* through the inactivation of *GSK3β* and subsequent inhibition of GLI2 ubiquitination, affecting GBM angiogenesis, metastasis, cell proliferation, and CSC’s regeneration [35]. GLI2 also influences both HH and Wnt pathways, playing a vital role in GBM stem cell (GSC) maintenance. GLI2 knockdown using lentiviral-mediated shRNA downregulates genes related to HH and Wnt signalling pathways, including leucine-rich repeat-containing GPCR 5, inhibits tumour cell proliferation and invasive capacity, and induces apoptosis [35].

Furthermore in the context of gliomas, the SHH pathway is associated with specific tumour grades. Studies demonstrate that the SHH pathway is active in grade II and III gliomas but not in grade IV de novo GBM’s. The pathway's activity and responsiveness are confined to progenitor cells within these tumours, suggesting a regulatory role in maintaining the proliferative and undifferentiated state of glioma progenitor cells. Abnormal activation of this pathway enhances the proliferative capability of grade II and III glioma cells, driving tumorigenesis [36, 37]. Further research has examined the expression of SHH pathway components in different glioma subtypes. Studies found higher expression levels of SHH-related genes in brainstem astrocytomas compared to supratentorial astrocytomas and normal brain tissue. This differential expression suggests that enhanced *PTCH1* expression and SHH pathway activation are involved in brainstem gliomas, potentially explaining the differences in malignant behaviour between brainstem and hemispheric gliomas. This indicates that the SHH pathway's role in gliomagenesis may vary significantly depending on the tumour's anatomical location and cellular context [38].

### Medulloblastoma

Medulloblastomas (MBs) are the most common malignant brain tumours in children, typically originating in the cerebellum. They are classified into four main molecular subgroups—WNT, SHH, Group 3, and Group 4—each with distinct prognostic and clinical implications [39, 40]. WNT-activated MBs have the most favourable prognosis, with five-year survival rates close to 100%, making them suitable candidates for reduced-intensity therapy to minimise long-term side effects [39]. SHH-activated MBs have variable outcomes influenced by genetic factors; TP53 mutations, for example, significantly worsen the prognosis, particularly in high-risk cases, which may require intensified treatment [41]. Additionally, other genetic markers like MYCN and GLI2 amplification affect outcomes in SHH MBs [41]. Group 3 MBs, comprising about 25% of cases, have the poorest prognosis, especially with MYC amplification, and a high metastatic rate (50%), necessitating aggressive treatment despite associated complications [42]. Group 4 MBs typically display an intermediate prognosis—better than Group 3 but less favourable than WNT—and span all age groups. They often show classic histology but may occasionally have large cell/anaplastic features, with poorer outcomes linked to FSTL5 expression [40, 43]. These molecular subtypes inform personalised therapy strategies, aiming to enhance survival rates and reduce treatment-related side effects. For instance, reduced-intensity therapy benefits WNT MBs, while high-risk SHH MBs or metastatic Group 3 MBs require more aggressive treatments tailored to their specific risk factors. This molecular classification has significantly advanced MB treatment, allowing for more precise, effective, and safer treatment planning [39, 43].

Similar to gliomas, dysregulation of the SHH signalling pathway has been implicated in MB pathogenesis [44]. Granule cells (GCs), the most abundant neurons in the cerebellum, are key to coordinating afferent inputs and motor outputs. During embryogenesis, granule cell precursors (GCPs) emerge from the upper rhombic lip and migrate to form the external granule layer (EGL) by embryonic day 13 (E13). Postnatally, these progenitors change shape and proliferate rapidly within the EGL, a process critical for proper cerebellar development. SHH, secreted by Purkinje cells (PCs), is a critical factor in GCP expansion, as evidenced by the fact that removal of PCs inhibits GCP proliferation and causes EGL thinning [45–47]. The most commonly mutated genes in the SHH pathway are *PTCH1* (44–45%), *SMO* (11–14%), *SUFU* (8–11%) and GLI2 (8–11%), leading to the sequential activation of GLI2, the downstream target of SHH signalling [48–50]. When *PTCH1* is inhibited, *SMO* initiates an intracellular signalling cascade that results in *GLI2* translocation to the nucleus, where it activates the transcription of target genes [51, 52]. *SUFU* acts as a negative regulator by repressing GLI activity, thereby affecting the production, trafficking and function of GLI proteins [53, 54]. When *PTCH1* is lacking in GCPs, it triggers the activation of the SHH signalling pathway, leading to abnormal proliferation and subsequent MB formation [55, 56]. This is supported by the study by Yang et al. in which knockout of *PTCH1* in mice resulted in the development of MB [56]. Similarly, forced activation of *SMO* in *PTCH1*-deficient mice resulted in hyperproliferation of GCPs, which ultimately led to a high incidence of MB formation [57, 58]. Loss of function of *SUFU* has been shown to lead to MB formation. Mutation of *SUFU* leads to the formation of truncated proteins that are unable to export the GLI transcription factor from the nucleus to the cytoplasm, resulting in activation of the SHH signalling pathway [59]. Math1-Cre-mediated deletion of *SUFU* in mouse GC precursors (GCPs) showed that deletion of *SUFU* resulted in both EGL hyperplasia and GCP proliferation [60].

The role of *GLI1* in MB remains controversial. Some studies report that silencing *GLI1* in MB cell lines leads to upregulation of target genes such as *PTCH1, cyclin D2, plakoglobin, NKX2.2* and *PAX6,* suggesting a positive role for *GLI1* in MB [61]. In addition, *Insm1* and *Nhlh1* have been identified as novel targets of HH signalling in the mouse cerebellum, with *Nhlh1* being directly activated by *GLI1* in cerebellar progenitor cells [62]. However, the role of *GLI1* appears to differ depending on the type of brain pathology. The researchers used retroviruses to inject the *SHH* gene into the developing brains of mouse embryos, activating the SHH pathway specifically in the cerebellum. This approach resulted in 76% of the mice developing MBs. Interestingly, GLI1, a transcription factor previously thought to be critical for SHH-induced tumourigenesis, was found to be non-essential for tumour formation, as MBs developed even in GLI1 null mutant mice [63].

*YAP1* has been identified as a critical effector in MB progression, and its up-regulation is associated with altered SHH signalling. *YAP1* plays an essential role in the proliferation of cerebellar granule neuron precursors (CGNPs), the cells of origin for certain MBs. It was localised in the nuclei of CGNPs and specifically in cells of the perivascular niche, where tumour-repopulating cells reside. Notably, *YAP1* was detected even in post-irradiation samples, suggesting its role in MB recurrence [64]. The interplay between *SHH* and insulin-like growth factor (IGF) signalling in MB formation has also been investigated. Using the RCAS/tv-a system to target the expression of *SHH, IGF2* and activated *Ak*t to nestin-expressing neural precursors in mice, the researchers found that co-expression of *SHH* with *IGF2* or *Akt* significantly increased tumour incidence. While *SHH* alone caused tumours in 15% of the mice, the combination of *SHH* with *IGF2* and *Akt* increased tumour incidence to 39% and 48%, respectively [65].

### Meningioma

SHH pathway aberrations are also evident in meningiomas, tumours arising from the meninges surrounding the brain and spine. The SHH pathway plays a regulatory role in cell proliferation and survival within these tumours. Recent large-scale genome sequencing studies have revealed that approximately 5% of meningiomas contain activating mutations in the SHH pathway, particularly in the SUFU gene. SUFU, a negative regulator of SHH signalling, is crucial for controlling pathway activation and is a potential therapeutic target. Studies emphasise the importance of genetic screening for SUFU mutations in families with a history of meningiomas [66, 67]. Further gene expression profiling studies have identified key genes involved in SHH pathway activation in meningiomas. Analysis of 36 meningioma specimens using real-time RT-PCR revealed 16 overexpressed genes, including *HHAT* and *DISP1*, which facilitate HH ligand release. *FOXM1*, a GLI transcription factor target, showed the highest mRNA level increase, particularly in aggressive tumours. Additionally, *SPP1* and *IGF2*, related to cell proliferation and extracellular matrix interactions, were notably overexpressed in higher-grade meningiomas [68].

### Neuroblastoma

Neuroblastoma (NB), a common paediatric cancer, originates from NCCs and can affect various parts of the body, including the brain and spine [69]. Within NBs, autocrine activation of the SHH pathway has been observed, where the tumour cells produce the SHH ligand themselves. Persistent activation of the SHH pathway in NB cells, indicated by high levels of SHH, PTCH1, SMO, and GLI2, suggests this autocrine mechanism. SHH binds to the PTCH1 receptor on the same cell, promoting continuous pathway activation, allowing pathogenic cells to evade apoptosis and proliferate. In addition, the SHH pathway actively influences neuroblastoma activity. Immunofluorescence staining reveals intact SHH signalling in NB cells, with SHH, PTCH1, GLI1 and GLI2 expressed in both the membrane and nucleus. This signalling pathway has a significant impact on NB by regulating cell proliferation, apoptosis, tumorigenicity and the cell cycle through modulation of CCND1 and p21 proteins. CCND1 facilitates progression from G1 to S phase of the cell cycle, while p21 induces G1 arrest and prevents entry into S phase by inhibiting cyclin-dependent kinases (CDKs) [70, 71]. In addition, hypoxia-inducible factor-1α (HIF-1α), which promotes proliferation, migration and invasiveness of NBs, does so through the SHH pathway. HIF-1α expression levels are strongly correlated with SHH, PTCH1 and GLI1, and GLI1 knockdown suppresses the effects of HIF-1α on NB proliferation, migration and invasiveness [72].

MicroRNAs (miRNAs) also play a role in modulating SHH signalling. The *miR181* family has been found to regulate the expression of *CDON*, a dependence receptor for SHH. This receptor promotes apoptosis in the absence of the SHH ligand. Studies have shown that high expression of *miR181* is tied to lower CDON levels and increased NB aggressiveness. *miR181* directly targets and degrades the 3′ UTR of CDON mRNA, leading to a reduction in protein levels. This miRNA-mediated regulation of *CDON* expression disrupts apoptotic signalling pathways, thereby promoting the survival and proliferation of NB cells [73].

### Chondroma

Chordoma is a rare, malignant bone tumour derived from embryonic notochordal remnants, typically occurring in the bones of the skull base and spine, most commonly the sacrum. These tumours are difficult to treat due to their proximity to vital structures, resistance to radiation, and unresponsiveness to conventional cytotoxic chemotherapy agents [74]. The SHH pathway, essential for chondrogenesis and involved in cellular differentiation, growth, and tissue patterning during embryonic development [75], remains active in certain pathological conditions, including various tumours. Limited studies have focused on the SHH pathway's role in chordoma formation and progression.

Research has utilised immunohistochemistry (IHC), genetic analysis, and in situ hybridization to detect SHH pathway components in cranial and spinal chordoma samples. These methods demonstrated overexpression of SHH and its downstream effectors, particularly *GLI1*, indicating active SHH signalling in chordomas. In contrast, these effectors are scarcely detectable in normal nucleus pulposus tissues [76]. RNA-Seq and NanoString analyses confirmed the upregulation of *PTCH1* and *GLI1*, further indicating SHH pathway activation in chordomas [77].

### Craniopharyngioma

Craniopharyngiomas (CPs) are rare, histologically benign but clinically aggressive tumours of the epithelium of Rathke’s pouch, primarily affecting the hypothalamic-pituitary axis. They are classified into two main types: adamantinomatous CPs (ACPs) and papillary CPs. The SHH signalling pathway plays a crucial role in cell differentiation and proliferation, such as in Rathke’s pouch, as well as in tumour cell migration [78].

Studies have investigated the role of SHH in CPs, detecting upregulation of SHH in human and mouse models [79]. In this model, SHH colocalizes in cells with nuclear accumulation of β-catenin, suggesting that both autocrine and paracrine SHH signalling contribute to ACP tumorigenesis. This hypothesis is supported by mRNA microarray gene expression analysis and targeted immunohistochemistry, which found overexpression of SHH in ACPs [78, 80]. In situ hybridization further confirmed significant expression of SHH pathway proteins, including SMO, GLI1, GLI3, and SUFU, indicating an active role of SHH signalling in promoting tumour cell proliferation and maintenance [81].

## Discussions and prospects

### The interplay between the SHH signalling pathway and other molecular signalling pathways in CNS tumours

#### With the WNT/beta-catenin signalling pathway

The SHH pathway plays a critical role in embryological processes such as cell proliferation, differentiation, and migration, mirroring its significance in oncogenesis. Studies underscore the shared molecular pathways regulating both normal development and tumour growth [82]. Initiation of the SHH canonical signalling pathway occurs when the SHH glycoprotein binds and deactivates *PTCH1*, releasing its inhibition on SMO, thereby indirectly regulating SMO activity [83]. Subsequent internalisation and degradation of the SHH-PTCH1 complex enable SMO activation through phosphorylation at the primary cilium [83]. Phosphorylated SMO translocates into the primary cilium, initiating downstream signalling cascades that culminate in nuclear translocation of GLI transcription factors and subsequent expression of GLI target genes [84].

The Wnt signalling pathway encompasses canonical and non-canonical routes, each with distinct mechanisms. Non-canonical Wnt pathways like the Wnt/Ca2 + pathway and planar cell polarity pathway function independently of β-catenin-T-cell factor/lymphoid enhancer-binding factor (TCF/LEF) [85]. In contrast, the canonical Wnt pathway (Wnt/β-catenin pathway) involves β-catenin translocation into the nucleus to activate target genes through TCF/LEF transcription factors, crucial for gene expression initiation [86]. This pathway consists of extracellular, membrane, cytoplasmic, and nuclear segments mediated by Wnt proteins (e.g., Wnt3a, Wnt1, Wnt5a), receptors (FZD, LRP5/6), and downstream components (β-catenin, DVL, GSK-3β, AXIN, APC, CK1), which regulate β-catenin levels and subsequent gene transcription [87, 88].

Implications of Wnt signalling in CNS tumours such as GBM, MB, meningioma, and pituitary adenomas are well-documented through complex pathways [48, 89–91]. In MB, interactions between SHH and Wnt signalling are evident. SHH signalling induces GLI1/2, promoting the expression of sFRP-1, which negatively regulates Wnt signalling by promoting cytoplasmic β-catenin accumulation [92]. Moreover, GLI1 activates Wnt ligands (Wnt2b, Wnt4, Wnt7b), stabilising β-catenin and enhancing Wnt pathway activation [93]. Both pathways converge on N-myc, a critical molecule in MB pathogenesis regulated by SHH through GSK3-β inhibition, promoting N-myc expression and stabilisation [94, 95]. GLI3 interaction with β-catenin C-terminal domain reduces Wnt-mediated transcriptional activity [96]. SUFU binds β-catenin, exporting it from the nucleus and repressing β-catenin/TCF-mediated transcription; SUFU loss leads to SHH and Wnt pathway overactivity, contributing to MB proliferation and differentiation failure [97].

In GBM, both canonical and non-canonical Wnt signalling pathways contribute to tumour proliferation and invasion by mimicking embryonic processes [98]. Elevated expression of canonical Wnt factors (WNT3A, TCF4) correlates with higher glioma grades and poor outcomes [99]. WNT/β-catenin signalling upregulates VEGF, supporting GBM angiogenesis [100]. Non-canonical Wnt factors (WNT5A, FZD-2) promote NSC proliferation and enhance neural differentiation, impacting GBM invasiveness [101, 102]. GLI1-mediated β-catenin stabilisation suggests a potential role of Wnt signalling in GBM development, warranting further investigation into the SHH-Wnt signalling relationship [103]. In summary, highlighting the intricate interactions between SHH and Wnt signalling pathways in CNS tumours is crucial for understanding their roles in tumorigenesis and identifying potential therapeutic targets.

#### Crosstalk with the Notch pathway

Notch signalling is a critical intercellular communication mechanism initiated by binding between a transmembrane receptor and ligands expressed on adjacent cells [104]. The Notch receptor precursor undergoes glycosylation in the endoplasmic reticulum (ER), affecting its affinity for various ligands. Following transport to the Golgi apparatus, proteolytic processing at S1 sites produces Notch extracellular subunit (NEC) and transmembrane/intracellular domain (NTMIC) heterodimers. These are then transported to the cell membrane as type I transmembrane proteins (NOTCH1-4 in mammals) [105, 106].

Interaction of the Notch receptor with canonical ligands (DLL1, DLL4, Jagged1, Jagged2) on neighbouring cells exposes a cleavage site hidden by the LNR domain. This triggers proteolytic cleavage at the S2 site by ADAM 10 or 17 enzymes, followed by γ-secretase-mediated cleavage within the endosome or at the plasma membrane, releasing the Notch intracellular domain (NICD) [107, 108]. NICD translocates to the nucleus where it binds with CSL, displacing the co-inhibitory complex and forming a NICD/CSL/Maml complex. This complex enhances the expression of downstream genes, including the *HES* family, which regulate proliferation and apoptosis [104, 109–111].

Notch signalling synergizes with SHH signalling during nervous system development [112] and plays a crucial role in oncogenesis, including CNS tumours such as gliomas, MBs, meningiomas, and choroid plexus papillomas [113, 114]. HES1, a downstream target of Notch signalling, modulates SHH signalling in glioblastoma by binding to N-boxes within *GLI1's* first intron, suppressing its expression and potentially inhibiting the HH cascade [115]. Mastermind-like1 (Maml1) regulates SHH signalling by interacting directly with GLI proteins, enhancing SHH-responsive gene expression [116]. Additionally, Jagged1, a Notch ligand, reduces *GLI2* expression, promoting apoptosis. Reciprocally, GLI2 downregulation reduces Jagged1 expression, highlighting crosstalk between SHH and Notch pathways [117]. Notch signalling also affects SHH signalling by promoting SHH-independent accumulation of SMO within the primary cilium, thereby influencing GLI3 activity and cilium length [118]. Moreover, the Notch pathway indirectly regulates SHH signalling through molecules such as Akt, STAT3, and mTOR, which promote stem cell survival [119]. While direct links to CNS tumours are less explored, these interactions suggest Notch signalling's role in promoting survival of CNS tumour cells.

In conclusion, Notch signalling's intricate regulation and crosstalk with SHH signalling contribute significantly to CNS tumour development and progression. Understanding these interactions provides insights into potential therapeutic targets for addressing dysregulated cell growth in CNS malignancies.

#### Relationship with the PI3K/AKT/mTOR pathway

The PI3K/AKT/mTOR (PAM) signalling pathway is pivotal in supporting tumour growth and progression by orchestrating cell cycle activities and regulating the synthesis of macromolecules such as proteins, nucleotides, and lipids [120, 121]. Within this pathway, mTOR exists in two distinct complexes: mTORC1 and mTORC2, each comprising mTOR along with Deptor and mLST8 subunits [122]. mTORC1, a component of the PAM pathway, becomes activated when growth factors stimulate PI3K, which subsequently activates AKT1. AKT1 then inhibits the tumour-suppressor TSC1/2 complex, releasing its inhibition on RHEB, thereby activating mTORC1 [123]. Activation of mTORC1 leads to the phosphorylation of ribosomal protein S6 kinase (S6K) and eukaryotic translation initiation factor 4E binding protein 1 (4E-BP1), which regulate mRNA translation, cell growth, and proliferation. Phosphorylation of 4E-BP1 prevents its binding to eukaryotic translation initiation factor 4E (eIF4E), crucial for initiating mRNA translation, while non-phosphorylated 4E-BP1 binds tightly to eIF4E, inhibiting translation [124–126].

**Fig. 2:**
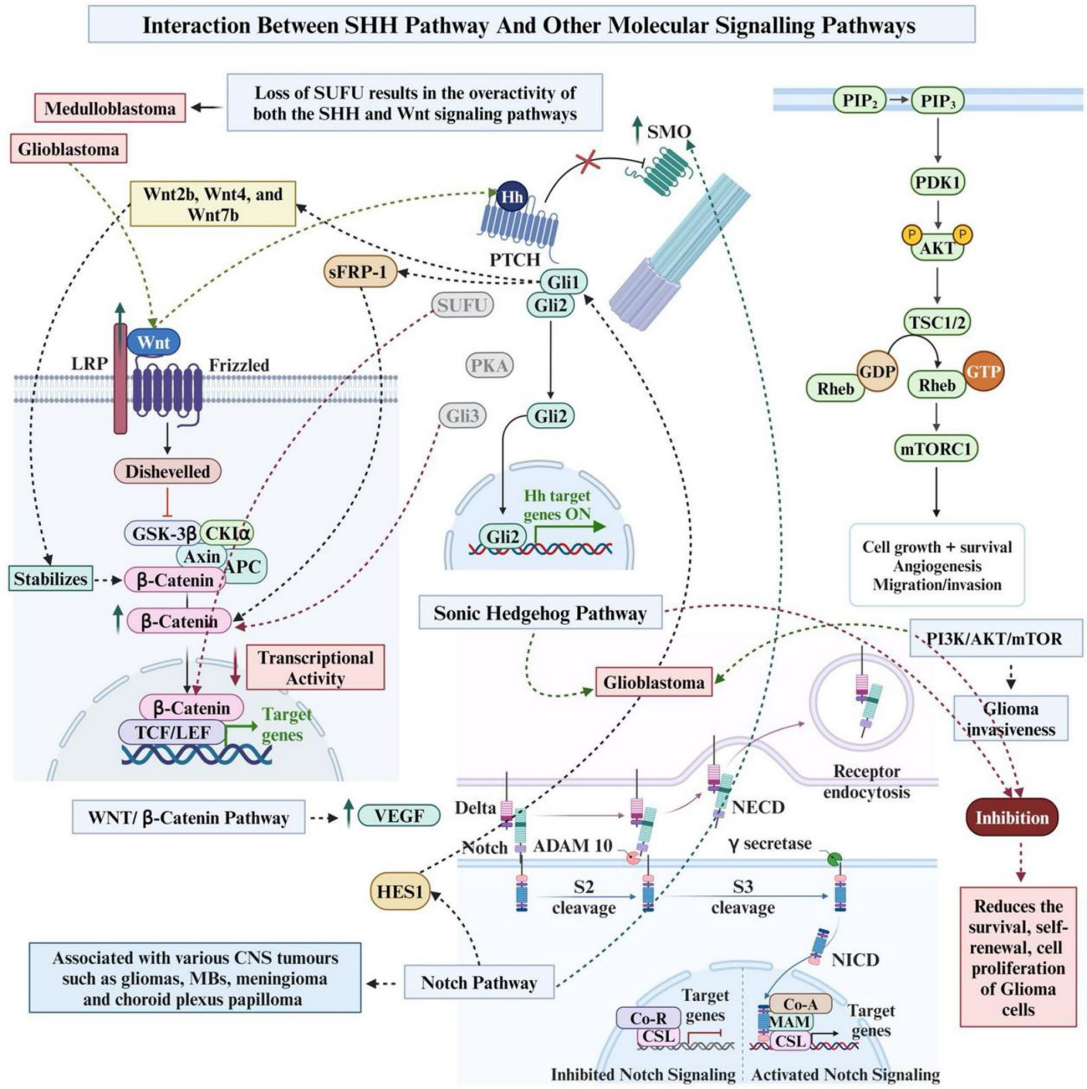
The interaction between the sonic hedgehog signalling pathway and other molecular pathways. Image was created with https://www.Biorender.com. SHH; Sonic Hedgehog, HH; Hedgehog, SUFU; Suppressor Of Fused, WNT; Wingless-Related Integrated Site, SMO; Smoothened, PTCH; Patched, GLI; Glioma Associated Oncogene, PKA; Protein Kinase A, sFRP; Secreted Frizzled-Related Protein, LRP; Low-Density Lipoprotein Receptor-Related Protein, GSK; Glycogen Synthase Kinase, APC; Antigen Presenting Cell, TCF; T-Cell Specific Transcription Factor, LEF; Lymphoid Enhancer-Binding Factor, VEGF; Vascular Endothelial Growth Factor, HES; Hairy And Enhancer Of Split, CNS; Central Nervous System, MB; Medulloblastoma, ADAM; A Disintegrin And Metalloproteinase, MAM; Mitochondrial-Associated Membranes, NICD; Notch Intracellular Domain, NECD; Notch Extracellular Domain, PIP; Phosphatidylinositol Phosphate, PDK; Pyruvate Dehydrogenase Kinase, AKT; Protein Kinase B, PI3K; Phosphatidylinositol 3-kinases, TSC; Tuberous Sclerosis Complex, Rheb; Ras Homolog Enriched In Brain, mTORC; Mammalian Target Of Rapamycin Complex, GDP; Guanosine Diphosphate, GTP; Guanosine Triphosphate

The RTK/PAM pathway is well-known for its role in enhancing the invasiveness of gliomas. Activation of mTOR by Akt phosphorylation leads to the activation of cyclin D1, which complexes with cyclin-dependent kinase (CDK) to drive the cell cycle from G1 to S phase, a critical step in promoting carcinogenesis when cyclin D1 is overexpressed. Additionally, Akt-mediated phosphorylation of P27kip1 neutralises its inhibitory effect on CDK activity, allowing for continued cell growth and differentiation [120, 121]. The interaction between SHH pathway and the PAM pathway has been demonstrated in various studies, particularly in GBMs. Studies on PTEN-deficient GBMs have shown that SHH and PI3K signalling pathways synergistically promote tumour growth and survival. Conversely, inhibiting both PI3K/Akt and SHH pathways results in tumour apoptosis and reduced growth of PTEN-deficient GBMs in experimental models, underscoring the crosstalk between these pathways [127]. Similarly, research by Nanta et al. demonstrates that blocking SHH and PAM pathways in GBM cells diminishes survival, self-renewal capacity, and expression of factors maintaining pluripotency, while also affecting cell proliferation and epithelial-mesenchymal transition [128]. Despite these insights, the precise mechanistic details of how SHH and the PAM pathway interact in CNS tumours remain unclear. However, studies on esophageal adenocarcinoma cells suggest that TNF-alpha activates GLI proteins through the mTOR pathway, specifically involving S6K1-mediated phosphorylation of GLI1 at Ser84 [129]. These findings provide mechanistic clues that similar interactions may occur in CNS tumours, highlighting the potential for therapeutic interventions targeting these pathways. Figure 2 summarises the interaction between SHH pathway and other molecular signalling pathways.

### The therapeutic relevance of SHH signalling pathways in CNS tumours

#### Drugs targeting SHH signalling pathway

The SHH signalling pathway plays a crucial role in the development and growth of various cancers. Consequently, numerous therapies targeting SHH signalling are emerging as promising neuro-oncological treatment strategies. Currently, drugs that target SHH signalling pathways in CNS tumours can be categorised into SMO inhibitors, GLI inhibitors and SHH inhibitors.

##### SMO inhibitors

Recent advancements in SHH pathway inhibitors, such as GDC-0449 and HhAntag, have sparked significant interest in their therapeutic potential. A clinical case study highlighted GDC-0449's efficacy in a 26-year-old patient with metastatic MB, leading to rapid tumour regression and symptom relief, albeit with transient response due to secondary mutations bypassing its effects [130]. Molecular insights revealed abnormal SHH pathway activation linked to PTCH1 gene loss, underscoring the need for personalised treatment approaches [130]. Combining GDC-0449 with HhAntag, which targets SMO via multiple binding sites, shows promise in overcoming drug resistance, with high-dose HhAntag demonstrating complete tumour eradication and prolonged MB-free survival [131].

Cyclopamine, acting on the PTCH-SMO complex, has demonstrated efficacy in reducing glioma, glioblastoma, and MB growth in mouse models and inhibiting human MB cell lines in vitro [132–134]. Recent studies revealed that Cyclopamine not only reduces cell viability but also enhances temozolomide (TMZ) efficacy by inducing apoptosis through cleaved caspase-3 activation, along with upregulating stemness markers like SOX-2 and OCT-4 in GBM cells [135]. Mechanistically, Cyclopamine disrupts the SHH signalling autocrine loop, downregulates BCL-2, and upregulates BAX, promoting apoptosis [70].

LDE-225 (Erismodegib/Sonidegib/Odomzo), a Cyclopamine derivative, effectively induces cell cycle arrest and apoptosis across various cancers, including GBM, by antagonising SMO and reducing GLI protein expression, validated in mouse models [136–138]. Approved for locally advanced basal cell carcinoma, LDE-225 is undergoing phase 2 trials for HH pathway-activated relapsed MB [139]. Conversely, vismodegib (GDC-0449), another SMO inhibitor, has shown mixed results, inhibiting chordoma growth but potentially enhancing tumour proliferation in certain contexts [23, 140].

Various next-generation inhibitors like IPI-926 (Saridegib), BMS-833923/XL139, PF-5274857, TAK-441, LY2940680 (Taladegib), and Itraconazole target different facets of SHH signalling. These compounds exhibit promising preclinical efficacy in SHH pathway-driven cancers, highlighting ongoing efforts to refine therapeutic strategies [141–147]. Itraconazole, for instance, synergizes with Cyclopamine in inhibiting HH-driven MB proliferation [146, 147]. These developments underscore the complexity and potential of SHH pathway inhibition in treating a spectrum of cancers, necessitating continued research into optimise therapeutic outcomes.

##### GLI inhibitors

GANT 61, a GLI antagonist, inhibits the DNA binding ability of GLI1 and GLI2. In vitro and in vivo studies have shown that GANT61 effectively reduces the expression of GLI1, c-MYC, MYCN, and Cyclin D1, leading to apoptosis in NB cells. Additionally, GANT61 enhances the efficacy of chemotherapeutic drugs used in NB treatment, either additively or synergistically, and suppresses the growth of established NB xenografts in nude mice [148].

##### SHH inhibitors

Gliomas pose significant challenges due to their aggressive nature; however, targeting the SHH pathway in GBM stem cells shows promise for therapeutic intervention. SHH signalling inhibitors effectively dismantle GBM cancer stem cells (CSCs) and prevent tumour recurrence by targeting SHH ligands, SMO, and GLI1 transcription factors at multiple points in the pathway [149]. Experimental models, such as zebrafish studies, have underscored the oncogenic potential of SHH signalling in the CNS. Activation of SHH signalling in zebrafish CNS models led to increased tumorigenesis, demonstrating the oncogenic role of SMOA1 in brain and retinal tumours [150].

In human tumour cells, inhibition of the SHH pathway reduces proliferation, highlighting its critical role in tumour growth [151]. In preclinical studies with orthotopic malignant glioma xenografts, pharmacological SHH pathway inhibition significantly improved survival rates by targeting CD133 + tumour-initiating cells responsible for tumour initiation and maintenance [152]. Despite initial success, tumours eventually regrew, suggesting the potential need for combination therapies for more effective treatment strategies. 5E1, a monoclonal antibody targeting the SHH ligand, inhibits MB growth in mouse models by preventing ligand binding to PTCH1. Treatment with 5E1 reduces tumour proliferation, promotes tumour cell apoptosis, and enhances survival rates compared to cyclopamine treatment [153]. Although these drugs await clinical approval for CNS tumours, they offer promising insights for future therapeutic approaches. Additionally, targeting SMO, GLI, and SHH signalling in non-CNS tumours suggests potential applications in CNS tumours, underscoring the need for further research. Furthermore, SHH signalling interacts with pathways like Wnt/β-catenin, Notch, and PI3K/Akt/mTOR, suggesting that combination therapies targeting these interactions could synergistically suppress tumour growth [154]. Comprehensive research into these interactions and treatments is crucial for advancing therapeutic strategies against CNS tumours.

##### DNA methyltransferase inhibitors

The expression of SHH pathway components and targets in NBs is regulated through both transcriptional and epigenetic mechanisms. Promoter regions of genes like PTCH1, HHIP, and SFRP1 can undergo methylation, impacting their expression levels. Studies indicate that hypermethylation of these promoters correlates with decreased expression of SHH pathway inhibitors, thereby enhancing pathway activation. This epigenetic modulation contributes to the aggressive nature of NBs. However, treatment with DNA methyltransferase inhibitors has shown potential in restoring expression of these epigenetically silenced genes, suggesting a therapeutic strategy for modulating SHH signalling in NBs [155]. Table 2 summarises the drugs targeting the SHH pathway in CNS tumours.

**Table 2:** Summary of drugs targeting the sonic hedgehog signalling pathway in central nervous system tumours

Therapeutic agents	Examples and functions
1. SMO inhibitors [130, 132–134, 136–138, 142–147]	GDC-0449 (Vismodegib): Inhibits SHH pathway, showing potential in treating metastatic MB HhAntag: Complements GDC-0449 by blocking SMO through additional binding sites Cyclopamine: Reduces growth rates of gliomas, GBMs, and MBs, enhances TMZ therapy by inducing apoptosis LDE-225 (Sonidegib): Induces cell cycle arrest and apoptosis, reduces epithelial-mesenchymal transition in multiple cancers IPI-926 (Saridegib): Suppresses tumour growth in MB allograft models BMS-833923: Decreases GLI1 and PTCH1 mRNA expression, inhibiting proliferation PF-5274857: Selective SMO antagonist, effective in MB allograft models TAK-441: Effective against Vismodegib-resistant SMO mutants LY2940680 (Taladegib): Inhibits HH signalling, effective against Vismodegib-resistant SMO mutants. Itraconazole: Inhibits SMO accumulation, effective in MB allograft models, synergistic with cyclopamine
2. GLI inhibitors [148]	GANT 61: Inhibits DNA binding of GLI1 and GLI2, downregulates GLI1, c-MYC, MYCN, Cyclin D1, induces apoptosis in NB cells, enhances effects of chemotherapeutic drugs
3. SHH inhibitors [153]	Target multiple components of the SHH signalling cascade, showing effectiveness in the breakdown of GBM cancer stem cells, preventing tumour recurrence - 5E1: Monoclonal antibody targeting SHH ligand, inhibits MB growth, reduces tumour proliferation, increases apoptosis, improves survival rates
4. DNA methyltransferase inhibitors [155]	Restore epigenetically silenced SHH pathway inhibitors, such as PTCH1, HHIP, and SFRP1, suggesting potential therapeutic approach for modulating SHH signalling in NBs

SMO; Smoothened, GLI; Glioma, SHH; Sonic Hedgehog, HH; Hedgehog, DNA; Deoxyribonucleic Acid, HhAntag; Hedgehog Signaling Antagonist, GBM; Glioblastoma, MB; Medulloblastoma, TMZ; Temozolomide, PTCH; Patched, mRNA; Messenger Ribonucleic Acid, SMO; Smoothened, c-MYC; Cellular Myelocytomatosis Oncogene, NB; Neuroblastoma, HHIP; Hedgehog-Interacting Protein, SFRP; Secreted Frizzled-Related Protein

#### Challenges with drug targeting the SHH signalling pathway in CNS tumour therapy

##### Genetic mutation

Genetic analysis of resistant tumours has revealed several mechanisms that confer resistance to SMO inhibitors like GDC-0449. These mechanisms include SMO mutations, SUFU loss, and amplification of GLI or HH target genes. In about 50% of resistant basal cell carcinomas (BCCs), SMO mutations maintain HH pathway activation despite inhibitor treatment. These mutations fall into two categories: those within the drug binding pocket (DBP) and those outside it (non-DBP). Mutations such as C469, D473, I408, V321, and W281 within the DBP impair SMO inhibitor binding [156, 157]. Specifically, the D473 mutation in SMO is associated with GDC-0449 resistance in MB cells by disrupting the drug's effective receptor binding. Studies using MB cells and allograft mouse models strongly support this, demonstrating how D473 mutations confer GDC-0449 resistance [158]. The D473Y mutation in vismodegib-resistant BCCs induces conformational changes in the binding site, disrupting the stabilising hydrogen bond network [159]. Computational docking studies identified other mutations like W281, V321, I408, and C469, which interfere with vismodegib binding. For instance, the SMO-W281C mutation disrupts the interaction critical for drug binding [156]. Mutations distal to the DBP, such as T241, A459, S533, and G497, may also confer resistance by destabilising SMO's architecture, promoting its activation and reducing inhibitor affinity [156, 157].

##### Non-canonical pathway activation

Initially, resistance was linked to mutations within the canonical HH pathway. However, subsequent research revealed non-canonical HH signalling pathways, such as AP-1 and TGF-β signalling, drive resistance by promoting Arhgef17 transcription. Arhgef17 activates RhoA, leading to actin polymerization and nuclear translocation of MRTF, enhancing GLI transcriptional activity independently of SMO inhibition [160, 161]. DYRK1B, a member of the DYRK family, influences HH signalling by regulating ligand expression and pathway activation via autocrine mechanisms. Inhibition of DYRK1B reduces GLI1 expression, offering a potential therapeutic target for GLI1-dependent cancers resistant to SMO inhibitors [162, 163]. Up-regulation of the insulin-like growth factor 1 receptor-phosphatidylinositol 3-kinase (IGF-1R-PI3K) signalling pathway correlates with increased PI3K expression in resistant MBs, contributing to resistance mechanisms [154]. Activation of the RAS/MAPK pathway, driven by mutations like HRAS (G12V) and BRAF (V600E), is significant in resistance and metastasis in HH-dependent cancers. These mutations enable cancer cells to proliferate independently of HH signalling, evading SMO inhibitors like LDE-225, GDC-0449, and LEQ-506 [164].

##### Loss of primary cilia

A newly discovered resistance mechanism involves the absence of primary cilia, conferring resistance to LDE-225 in MB cells. Primary cilia, crucial for HH pathway signal transmission, are lost during tumour development, unexpectedly shielding tumour cells from SMO inhibitors [165]. Genome-wide transposon mutagenesis screening in HH-dependent MB cells identified SUFU and oral-facial-digital syndrome 1 (OFD1) genes as critical in this resistance mechanism. Mutations in *OFD1* lead to cilia loss, resulting in slow-growing, GLI2-dependent resistant tumours. In cilia-deficient cells, only the full-length form of GLI2 (GLI2-F) is present, unaffected by SMO inhibitors. The absence of cilia disrupts GLI2 proteolytic processing, preventing the formation of the truncated repressor form (GLI2-R). Consequently, HH signalling remains active with unprocessed GLI2-F, allowing cilia-deficient cells to evade drug inhibition [165].

##### Adverse reactions

Another challenge in developing drugs targeting the SHH signalling pathway is the potential for adverse reactions or side effects. Currently, only a few drugs targeting the SHH pathway—namely GDC-0449, LDE-225, IPI-926, LY2940680, and TAK-441—have documented adverse effects. GDC-0449 has been associated with muscle cramps, taste disturbances, weight loss, hair loss, and weakness [166]. LDE-225 side effects include muscle spasms, taste disorders, nausea, alopecia, and elevated creatine kinase levels. Although these reactions are often mild, long-term adverse effects can significantly impact patients' quality of life and lead to drug withdrawal [167]. Compared to GDC-0449, patients receiving LDE-225 reported fewer and slower-occurring adverse events [168]. IPI-926, LY2940680, and TAK-441 commonly cause fatigue, nausea, and muscle spasms, with liver dysfunction and alopecia specific to IPI-926 [169–171]. The adverse reactions of other drugs targeting the SHH pathway have not yet been identified due to the lack of clinical trials. Therefore, further research is crucial to uncover potential side effects and deepen our understanding of the safety profiles of these therapies, ultimately improving patient care and outcomes.

#### Strategies for overcoming resistance to SMO inhibitors

Several strategies have been proposed to overcome resistance to drugs targeting the SHH signalling pathway. One approach involves the development of second-generation SMO inhibitors. HH003, a novel SMO inhibitor featuring a tetrahydropyrido(4,3-d)pyrimidine scaffold, has demonstrated efficacy in blocking the SHH pathway by suppressing the transcription of target genes such as *GLI1* and *PTCH1*, induced by pathway agonists. Both in vitro and in vivo studies have confirmed the anti-tumor activity of HH003, effectively inhibiting the growth of various cancer cells, including glioblastoma T98G and SF295 [172]. Another promising strategy entails the use of the Bcl-2 homology 3 mimetic ABT-199, which can overcome resistance caused by SMO mutations. ABT-199 suppresses SHH signalling by acting as a competitive inhibitor of oxysterol, likely targeting the cysteine-rich domain of SMO. It has effectively reduced SMO agonist (SAG)-stimulated HH activity in Light II cells with various SMO mutants. In MB transgenic mice harbouring the SMO-W539L mutation, ABT-199 significantly inhibited tumour growth, whereas GDC-0449 showed no effect, suggesting that ABT-199 can overcome resistance to current SMO inhibitors caused by SMO mutations [173].

Additionally, combination therapies offer a viable strategy to combat resistance. For instance, combining AMPK activators with Vismodegib can overcome Vismodegib resistance and inhibit the growth of SMO-D473G MB cells. In both mouse subcutaneous and intracranial models, the combination of Metformin and Vismodegib showed synergistic suppression of MB tumour growth [174]. Similarly, combining LDE-225 with the PI3K class I inhibitor NVP-BKM120 or the dual PI3K/mTOR inhibitor NVP-BEZ235 has markedly delayed the development of resistance in MB tumours derived from PTCH^+ /−^ p53^−/−^ mice [154]. Moreover, the combination of Itraconazole and ATO has significantly improved anti-tumor efficacy in a subcutaneous allograft model of PTCH^+/−^ ;p53^−/−^ mice. Itraconazole inhibited the activity of all known SMO resistance mutants at levels similar to SMO-D477G, resulting in inhibited tumour growth and reduced tumour volumes in SMO-resistant tumours [147]. These findings suggest that combination therapy may represent the future direction for overcoming resistance to SHH pathway-targeting drugs. Further research is essential to identify the most effective combinations and to develop new drugs that can enhance treatment efficacy and improve patient outcomes. The challenges of targeting the SHH pathway in CNS tumour therapy and strategies to overcome resistance to SMO inhibitors are summarised in Table 3.

**Table 3:** Summary of challenges with drug targeting in the sonic hedgehog signalling pathway in central nervous system tumour therapy and strategies for overcoming resistance to smoothened inhibitors

Challenges/strategies	Description
Challenges	
Genetic mutations [156–158]	Analysis of resistant tumours has revealed various genetic mutations that cause resistance to SMO inhibitors like GDC-0449. These include mutations in SMO, loss of SUFU, and amplification of GLI or HH target genes. Around 50% of resistant BCCs have SMO mutations that continue to activate the HH signalling pathway despite treatment. These mutations can occur in the DBP or other regions, affecting drug binding and causing resistance
Non-canonical pathway activation [154, 160–164]	Resistance can also result from non-canonical HH signalling pathways, such as AP-1 and TGF-β, which enhance GLI transcriptional activity independently of SMO inhibition by promoting Arhgef17 transcription. DYRK1B regulates ligand expression and pathway activation via autocrine mechanisms. Increased IGF-1R-PI3K signalling pathway activity and RAS/MAPK pathway activation are also linked to resistance, enabling cancer cells to grow independently of the HH signalling pathway and evade SMO inhibitors
Loss of primary cilia [165]	Another mechanism of resistance involves the loss of primary cilia, which contain key components of the HH pathway. Cilia loss during tumour development protects tumour cells from SMO inhibitors. Mutations in the *OFD1* gene lead to cilia loss, resulting in tumours dependent on GLI2 activity. In cells without cilia, only the full-length form of GLI2 (GLI2-F) remains, ensuring continuous, low-level HH signalling activity and allowing cells to evade drug effects
Adverse reactions and side effects [166, 168–170]	Targeting the SHH signalling pathway with drugs can cause adverse reactions or side effects. Known side effects for drugs like GDC-0449, LDE-225, IPI-926, LY2940680, and TAK-441 include muscle cramps, taste disturbances, weight loss, hair loss, weakness, muscle spasms, nausea, alopecia, and elevated creatine kinase levels. Although often mild, these reactions can significantly impact patients' quality of life and lead to drug discontinuation. Further research is needed to identify potential side effects and improve understanding of these therapies' safety profiles
Strategies for overcoming resistance	
Second-generation SMO inhibitors [172]	HH003, a new SMO inhibitor with a tetrahydro-pyrido(4,3-d)pyrimidine scaffold, effectively blocks the SHH pathway by suppressing the transcription of target genes like GLI1 and PTCH1. In vitro and in vivo studies have shown that HH003 inhibits the growth of various cancer cells, including glioblastoma T98G and SF295, making it a promising second-generation SMO inhibitor
Bcl-2 homology 3 mimetic ABT-199 [173]	ABT-199 overcomes resistance caused by SMO mutations by acting as a competitive inhibitor of oxysterol, likely targeting the cysteine-rich domain of SMO. It effectively reduces SMO agonist (SAG)-stimulated HH activity in Light II cells with various SMO mutants. In MB transgenic mice with the SMO-W539L mutation, ABT-199 significantly inhibited tumour growth, indicating its potential to bypass resistance to current SMO inhibitors due to SMO mutations
Combination therapy [147, 154, 174]	Combining AMPK activators with Vismodegib can overcome resistance and inhibit the growth of SMOD473G MB cells. In mouse models, the combination of Metformin and Vismodegib showed synergistic suppression of MB tumour growth. Additionally, combining LDE-225 with PI3K class I inhibitor NVP-BKM120 or dual PI3K/mTOR inhibitor NVP-BEZ235 delayed resistance development in MB tumours. The combination of Itraconazole and ATO also improved anti-tumor efficacy in SMO-resistant tumours. These combination therapies have shown promise in inhibiting tumour growth and reducing tumour volumes

SMO; Smoothened, GLI; Glioma, HH; Hedgehog, SUFU; Suppressor Of Fused, BCC; Basal Cell Carcinoma AP; Activator Protein 1,TGF-B; Transforming Growth Factor Beta, DYRK1B; Dual Specificity Tyrosine-Phosphorylation-Regulated Kinase 1B, IGF; Insulin-Like Growth Factor, PI3K; Phosphatidylinositol 3-Kinase, MAPK; Mitogen-Activated Protein Kinase, OFD; Orofaciodigital syndrome type 1, PTCH; Patched, SAG; Smoothened Agonist, AMPK; Activated Protein Kinase, MB; Medulloblastoma, ATO; Arsenic Trioxide, mTOR; Mammalian Target of Rapamycin

### Novel biomarkers for SHH signalling pathway activation in CNS tumours

Biomarkers are crucial for identifying and stratifying CNS tumours such as MBs and meningiomas. GAB1 has emerged as a significant biomarker, detectable through immunohistochemistry and useful in excluding HH-independent meningiomas [175]. Additionally, GAB1 serves as a diagnostic marker for SHH tumours, shown by specific reactivity of anti-GAB1 antibodies [176]. In SHH MB, Shih et al. developed a risk stratification scheme categorising patients into high-risk, standard-risk, and low-risk groups based on biomarkers. High- and standard-risk patients are identified by GLI2 amplification, 14q loss, and leptomeningeal dissemination, with GLI2 amplification alone correlating with poor prognosis. Absence of these markers defines a low-risk group similar to WNT tumour patients, highlighting GLI2 and 14q loss as reliable prognostic indicators [177].

YAP1 has emerged as a predictive marker for response to SMO inhibitors in SHH MB patients. Those genetically resistant to SMO inhibitors, particularly in the aggressive alpha subtype, often exhibit YAP1 overexpression. SHH-like cell lines with TP53 mutations show enhanced SMO inhibitor responsiveness upon YAP1 depletion, suggesting YAP1 as a therapeutic target to improve outcomes [178]. Combining Sonidegib and Verteporfin, which inhibit SMO and YAP1 respectively, shows synergistic effects, proposing a dual inhibition strategy for overcoming resistance in SHH MB therapy [178]. *GLI1* is another promising biomarker, particularly for predicting neuroblastoma (NB) severity with high MYCN expression. MYCN amplification is associated with more aggressive NB. GLI1-positive NB cases without MYCN amplification correlate with early clinical stages and improved outcomes, while low *GLI1* expression and MYCN amplification correlate with advanced disease and poor prognosis. Only 10% of MYCN-amplified cases were positive for *GLI1*, suggesting *GLI1* expression as a biomarker for NB differentiation and prognosis [179].

Further research is necessary to identify additional biomarkers and ensure they are accessible, cost-effective, and reliable in terms of specificity and sensitivity. This will enhance their clinical utility for diagnosis, prognosis, and treatment decisions.

## Study limitations

There are several important limitations that must be addressed in this review. Firstly, the majority of studies included are preclinical, providing valuable insights into the mechanisms and implications of SHH signalling and its interplay with other molecular pathways in CNS tumours. However, the clinical implications of these findings remain largely speculative at this stage. Secondly, many of the drugs discussed are primarily studied in contexts other than CNS tumours, making it challenging to draw definitive clinical conclusions. While some clinical trials have been conducted with these drugs in CNS tumour settings, many have either been terminated early or completed only up to phase 2, resulting in limited understanding of their mechanisms, efficacy, safety profiles and adverse reactions specifically in CNS tumours. Furthermore, certain studies have explored drugs not approved for CNS tumours, yielding promising results, but often with small patient cohorts that may not be representative of the broader population.

The complexity of the SHH signalling pathway and its interactions with other pathways in CNS tumours remains poorly understood, necessitating further research to bridge these knowledge gaps. Given the dynamic nature of SHH interactions with various cellular pathways, this review may not comprehensively cover all recent discoveries or emerging insights into how these interactions influence disease progression and therapeutic responses. Additionally, the diverse characteristics among different cancer types and within CNS tumours pose challenges in fully capturing their heterogeneity, potentially limiting the applicability of findings across all clinical contexts. Addressing these limitations will not only enhance our understanding of the SHH signalling pathway and its therapeutic implications in CNS tumours but also facilitate the development of more effective treatments that leverage cellular regulatory mechanisms and interactions with other molecular signalling pathways, ultimately improving clinical outcomes.

## Conclusion

In conclusion, The SHH signalling pathway plays a crucial role in the proliferation and growth of certain CNS tumours. Understanding the molecular mechanisms of SHH signalling is essential for developing improved therapeutic strategies that suppress this pathway and enhance treatment outcomes. Future research should focus on exploring combination therapies, identifying new molecular targets, and potentially exploring the benefits of gene editing technologies to optimise treatment modalities for patients with CNS tumours. Additionally, investigating genetic variations within SHH signalling tumours can provide valuable insights for personalised medicine, allowing for tailored treatments and reducing the risk of resistance. A greater emphasis should be placed on understanding the interplay between SHH signalling and other molecular pathways in CNS tumours including aspects of the tumour immune microenvironment. This knowledge is critical for unlocking the full therapeutic potential of medication-directed therapy and translating these findings into clinical practice. By addressing these areas, we can move closer to developing more effective and personalised treatments for SHH-related CNS tumours, ultimately improving patient outcomes.

## Data Availability

Not applicable.

## Abbreviations

HH: Hedgehog
SHH: Sonic Hedgehog
CNS: Central nervous system
IDH: Intradialytic hypotension
TERT: Telomerase reverse transcriptase
TREM: Triggering receptor expressed on myeloid cells
TME: Tumour microenvironment
PTCH: Patched
SMO: Smoothened
NT: Neural tube
NTD: Neural tube development
NSC: Neural stem cells
NPC: Neural progenitor cells
PC: Purkinje cell
GCP: Granule cell progenitor
KIF: Kinesin family member
SUFU: Suppressor of fused
GBM’s: Glioblastoma multiformes
CTD: C-terminal domain
CSC’s: Cancer stem cells
CGNP’s: Cerebellar granule neuron precursors
IGF’s: Insulin-like growth factors
YAP: Yes-associated protein
GB: Glioblastoma
NB: Neuroblastoma
MB: Medulloblastoma
ISX: Isoxazole
HDAC’s: Histone deacetylases
miRNA: Micro-ribonucleic acid
IHC: Immunohistochemistry
RFS: Recurrence-free survival
OS: Overall survival
CP’s: Craniopharyngiomas
ACP’s: Adamantinomatous craniopharyngiomas
MB: Medulloblastoma
EGL: External granule layer
GPCR: G-protein coupled receptor
TCF/LEF: T-cell factor/lymphoid enhancer-binding factor
CK: Casein kinase
RCAS: Replication-competent avian sarcoma
TVA: Tumor virus A
APC: Adenomatous polyposis coli
GSK: Glycogen synthase kinase
FZD: Frizzled
PNET: Primitive neuroectodermal tumor
VEGF: Vascular endothelial growth factor
ER: Endoplasmic reticulum
CDON: Cell adhesion molecule-related/downregulated by oncogenes
NEC: Notch extracellular subunit
NEXT: Notch extracellular truncation
NICD: Notch intracellular domain
MAML: Mastermind-like
TMZ: Temozolomide
sFRP: Secreted frizzled-related protein
PAX: Paired box protein
INSM: Insulinoma
NHLH: Nescient helix-loop-helix
HHAT: Hedgehog acyltransferase
DISP: Dispatched
FOXM: Forkhead box M
SPP: Secreted phosphoprotein
PCR: Polymerase chain reaction
HDAC: Histone deacetylase
BCC: Basal cell carcimoa
PNET: Primitive neuroectodermal tumour
CAF: Cancer-associated fibroblast
rhSHH: Recombinant human Sonic hedgehog
FLT: Fms-related tyrosine kinase
MMP: Matrix metalloproteinase
